# Frequency-dependent effects of 0.05% atropine eyedrops on myopia progression and peripheral defocus: a prospective study

**DOI:** 10.1186/s40662-024-00395-0

**Published:** 2024-08-01

**Authors:** Yuanfang Yang, Minsong Xue, Jiangdong Hao, Zhenghua Lin, Xiaoyun Xi, Haoran Wu, Longbo Wen, Qinglin Xu, Zhiwei Luo, Guangyao Ran, Pablo Artal, Weizhong Lan, Xiaoning Li, Zhikuan Yang

**Affiliations:** 1 Aier School of Ophthalmology, Central South University, Changsha, 410015 China; 2 Changsha Aier Eye Hospital, Changsha, 410015 China; 3 School of Stomatology and Ophthalmology, Xianning Medical College, Hubei University of Science and Technology, Xianning, 437000 China; 4 Aier Institute of Optometry and Vision Science, Changsha, 410015 China; 5 Hunan Province Optometry Engineering and Technology Research Center, Changsha, 410015 China; 6 Hunan Province International Cooperation Base for Optometry Science and Technology, Changsha, 410015 China; 7 Laboratorio de Óptica, Universidad de Murcia, Campus de Espinardo, 30100 Murcia, Spain

**Keywords:** 0.05% atropine eyedrops, Myopia control, Peripheral defocus, Medication compliance

## Abstract

**Background:**

Atropine, specifically 0.05% eyedrops, has proven effective in slowing myopia progression. This study aims to investigate peripheral refraction (PR) characteristics in myopic children treated with 0.05% atropine eyedrops at different frequencies.

**Methods:**

One hundred thirty-eight myopic children completed this one-year prospective study, randomly assigned to once daily (7/7), twice per week (2/7), or once per week (1/7) groups. Spherical equivalent (SE) and axial length (AL) were measured. PR was assessed using a custom-made Hartmann-Shack wavefront peripheral sensor, covering a visual field of horizontal 60° and vertical 36°. Relative peripheral refraction (RPR) was calculated by subtracting central from peripheral measurements.

**Results:**

After one year, SE increased more significantly in the 1/7 group compared to the 7/7 group (*P* < 0.001) and 2/7 group (*P* = 0.004); AL elongation was also greater in the 1/7 group compared to the 7/7 group (*P* < 0.001). In comparison with higher frequency groups, 1/7 group exhibited more myopic PR in the fovea and its vertical superior, inferior, and nasal retina; and less myopic RPR in the periphery retina after one-year (*P* < 0.05). Additionally, RPR in the 7/7 group demonstrated myopic shift across the entire retina, the 2/7 group in temporal and inferior retina, while the 1/7 group showed a hyperopic shift in the superior retina (*P* < 0.05). Moreover, myopic shift of RPR in the temporal retina is related to less myopia progression, notably in the 7/7 group (*P* < 0.05).

**Conclusions:**

Atropine inhibits myopia progression in a frequency-dependent manner. The once-daily group showed the slowest myopia progression but exhibited more myopic shifts in RPR. Additionally, RPR in the temporal retina was related to myopia progression in all groups.

**Trial registration:**

Chinese Clinical Trial Registry, ChiCTR2100043506. Registered 21 February 2021, https://www.chictr.org.cn/showproj.html?proj=122214

**Supplementary Information:**

The online version contains supplementary material available at 10.1186/s40662-024-00395-0.

## Background

Myopia is a growing health concern in East and Southeast Asia given its escalating prevalence, reaching 80%–90% among high school graduates [1]. About 10%–20% of them develop high myopia, associated with sight-threatening ocular complications [2]. Among interventions targeting axial length (AL), atropine, orthokeratology, peripheral defocus-modifying contact lenses, pirenzepine, and progressive addition spectacle lenses are effective, with pharmacological methods such as atropine proving most efficacious [3]. Higher doses are more effective in inhibiting myopia progression [4]. Yam et al. confirmed that nightly use of 0.05% atropine eyedrops significantly reduces myopia incidence and rapid myopic shifts [5]. Consequently, the optimal concentration for myopia prevention and control is currently determined to be 0.05% atropine, balancing efficacy and side effects [6, 7]. Weekly atropine use, similar to daily administration for amblyopia, yields comparable vision improvement [8], prompting exploration of less frequent application for achieving similar myopia control.

The exact site and mechanism of atropine in preventing myopia remains unclear [9, 10]. To explore whether defocus may influence the observed treatment effects, we need to explore peripheral refraction (PR) with atropine. Animal models indicate that eliminating fovea signals does not interfere with emmetropization or lens-induced hyperopic defocus in the peripheral retina but still promotes axial myopia [11, 12]. Mutti et al. observed more hyperopic relative peripheral refraction (RPR) two to four years before myopia onset, with more stable RPR changes after onset [13]. Corrective modalities inducing relative peripheral myopia, both spectacle and contact lens, have achieved success in myopia control [14, 15]. Studies demonstrated that 0.01% atropine can reduce relative hyperopia in the temporal retina and the hyperopic shift from cycloplegia in myopic children [16]. Notably, myopic children treated with atropine exhibit significant differences in RPR compared to non-atropine groups [17]. However, the role of PR in the development of myopia remains contentious. Some studies suggest that relative peripheral hyperopia does not reliably indicate the onset or advancement of myopia in children [18, 19]. Therefore, considering daily dosing as the standard, we used the once-daily dosing group as the reference to comprehensively investigate the relationship between atropine use and PR.

This study aimed to investigate changes in PR and RPR among myopic children using 0.05% atropine eyedrops of different frequencies over one year. We utilized a custom-made Hartmann-Shack wavefront peripheral autorefractor (Voptica Peripheral Refraction, Voptica SL) [20, 21] to assess PR, covering a horizontal field of 60° (from temporal 30° to nasal 30°) and a vertical field of 36° (from superior 20° to inferior 16°). Additionally, we explored whether RPR induced by 0.05% atropine eyedrops was associated with changes in refraction and AL over one year.

## Methods

### Study population

In total, 155 Asian children were enrolled in Changsha Aier Eye Hospital (Changsha, China) between October 2021 and September 2022. Finally, 138 children completed all follow-up examinations. Detailed information on study dropouts is shown in Additional file 1. This study was approved by the hospital’s Committee of Research Ethics (ID: 2020KYPJ001) and conducted following the tenets of the Declaration of Helsinki. The study was registered with the Chinese Clinical Trial Registry (ChiCTR2100043506). Each participant and one of their parents signed an informed consent form after receiving a detailed explanation of the procedures and risks involved.

Inclusion criteria were children who were (1) aged 6–14 years; (2) had a refractive error of spherical equivalent (SE) between − 0.50 D and − 5.00 D in both eyes, astigmatism of 2.00 D or less in both eyes, best-corrected distance visual acuity of 0.1 logMAR or better in both eyes and intraocular pressure ≤ 21 mmHg; and (3) had no history of using other myopia control lenses or medications. Exclusion criteria included children with other combined ocular diseases (e.g., amblyopia, strabismus, corneal scar, cataract, glaucoma, or ocular tumor), prior myopia control interventions, or allergies to atropine, cyclopentolate, or excipients.

### Intervention

The 0.05% atropine solution used in this study was prepared in the prescription room of Changsha Aier Eye Hospital. Participants were randomly allocated to three groups: once-daily group (7/7 group, 47 subjects), using 0.05% atropine nightly; twice per week group (2/7 group, 49 subjects), using 0.05% atropine on fixed nights twice per week; and once per week group (1/7 group, 42 subjects), using 0.05% atropine once weekly. All subjects received 0.05% atropine eyedrops for both eyes continuously for one year based on their subgroup.

Two research assistants assessed compliance through oral inquiries and by checking the medication administration record book provided by the parents. Additionally, all subjects were instructed to wear single-vision spectacle lenses in conjunction with atropine use. In our study, both the researchers responsible for grouping and the participants were fully informed. However, to minimize potential biases, during the experimental procedures, the examiners responsible for conducting the examinations and assessments remained blinded to participants' conditions.

### Main outcome measures

PR in the right eye was assessed utilizing a custom open-view Hartmann-Shack wavefront sensor (Voptica Peripheral Refraction, VPR, Voptica SL, Murcia, Spain). Measurements were conducted on naked right eyes without cycloplegia after a 30-min adaptation in a dark room. The instrument, detailed in previous publications [20, 21], featured a motorized optical arm scanning 60° of the horizontal visual field in 1° increments over 1.3 s. Refractive maps were generated from four scans and averaged for analysis. Vertical refraction was measured using fixation targets positioned 2.5 m away, with 10 cross-shaped lighting targets manually controlled by the operator. The top target corresponds to superior 20° and the lowest target to inferior 16°. The interval was 4° for adjacent targets. The two-dimensional (2D) refractive maps generated from 10 horizontal scans (610 data collection points) were produced using spline-based interpolation. Refraction analysis was confined to the central 4-mm pupil area. For each retinal location, the mean of the four measurements at each retinal location was used, and refraction was calculated from the second-order terms, expressed as the SE refractive error (spherical power + 1/2 cylindrical power).

Central SE of the subject’s cycloplegic autorefraction was determined using an auto refractometer (ARK-510A, Nidek, Japan). Cycloplegia was induced with 3 drops of tropicamide (Mydrin P; Santen, Osaka, Japan), with a 5-min interval between drops. Adequate cycloplegia was confirmed after 30 min if the pupillary light reflex was absent or pupil size exceeded 6.0 mm. AL was measured using LENSTAR (LS 900, Haag-Streit AG, Koeniz, Switzerland) before the induction of cycloplegia. These measurements were taken both at the baseline and the one-year follow-up.

### Data processing and statistical analyses

**Fig. 1:**
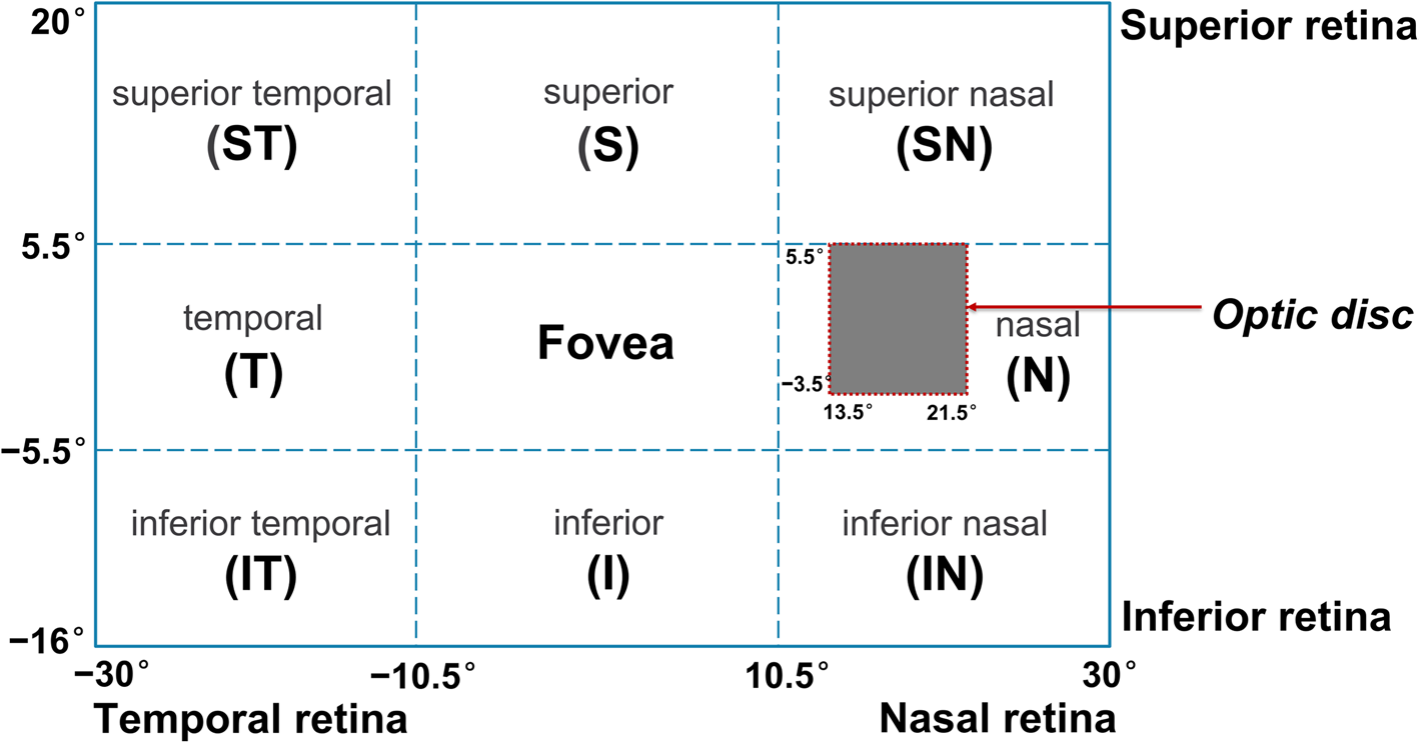
Codes used for the different zones in the statistical analysis. The red dotted lines represent the optic disc areas excluded from the analysis. The x-axis coordinates of the map show the horizontal meridian, where positive angles correspond to the nasal retina (temporal visual field) and negative angles to the temporal retina (nasal visual field). On the y-axis, positive angles indicate the superior retina (inferior visual field) while negative angles indicate the inferior retina (superior visual field)

2D maps of the right eye were generated from 10 horizontal Sections. (610 points in the retina) using custom MATLAB scripts (MathWorks, Natick, MA, USA). Positive angles indicated the nasal retina while negative angles indicated the temporal retina in abscissas. In the ordinates, positive angles indicated the superior retina and negative angles indicated the inferior retina. To compare the differences in PR and RPR between groups, the 2D maps were divided into 3 × 3 regions, with the mean value of each zone used for statistical analysis. Figure 1 illustrates spatial analyses with segmentation at superior 5.5° and inferior 5.5° horizontally, and nasal 10.5° and temporal 10.5° vertically, excluding data points around the optic disc (13.5 < x < 21.5, − 3.5 < y < 5.5). The selection of these zones was informed by results and outcomes from our previous studies [20, 22].

Data from each participant’s right eye were analyzed using SPSS commercial software (version 26.00, IBM, Armonk, NY, USA). Normally distributed data were expressed as mean ± standard deviation (SD). One-way analysis of variance (ANOVA) was utilized to compare age, SE, AL, PR, RPR, and one-year changes across the three groups. Post hoc analysis for multiple comparisons was performed using Bonferroni tests. The Chi-squared test was used to assess sex distribution differences among groups. Additionally, a paired t-test was utilized to compare the one-year longitudinal changes in average refractions within each group across different retinal areas. Pearson’s correlation analysis was utilized to explore relationships between RPR changes in each zone and changes in SE or AL. Comprehensive statistical methods have been provided in Additional file 2. Two-tailed *P* < 0.05 was considered statistically significant.

## Results

### Baseline demographic and myopia progression

**Fig. 2:**
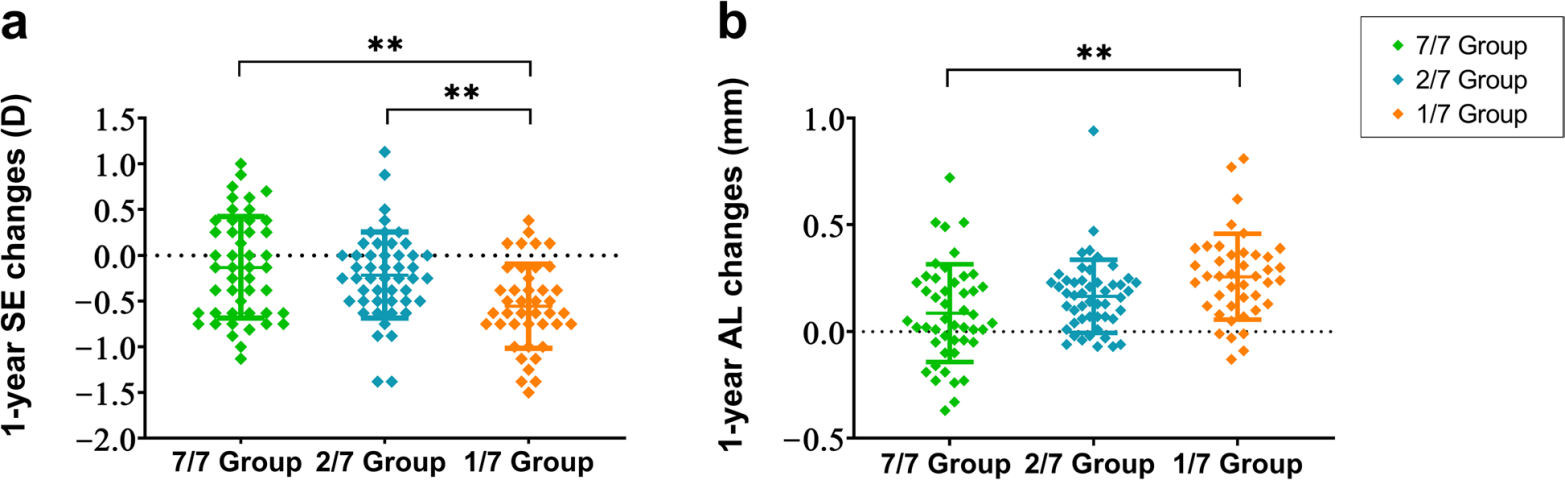
One-year spherical equivalent (SE) and axial length (AL) changes among the three different atropine regimen groups. 7/7 Group: once daily, 2/7 Group: twice per week, 1/7 Group: once per week. **a** One-year SE changes; **b** One-year AL changes. ** *P* < 0.01

A total of 138 children (138 eyes) completed all examinations and the one-year follow-up visit. Demographic and myopia progression data at the baseline and one-year follow-up are presented in Table 1 and Fig. 2. At baseline, there were no statistically significant differences in age, sex, SE, and AL among the three groups (all* P* > 0.05). After one year of 0.05% atropine eyedrop usage, notable differences in the increase of SE were observed between the 1/7 group and the other two groups. Additionally, significant differences in AL elongation were noted between the 1/7 and 7/7 groups (*P* < 0.001). Concerning side effects associated with atropine usage, only one patient in the 7/7 group experienced blurred vision, resulting in discontinuation, as indicated in Additional file 1.

**Table 1:** Baseline demographic and myopia progression data (mean ± SD)

Parameters	7/7 Group	2/7 Group	1/7 Group	^#^ *P* value	7/7 vs*.* 2/7	7/7 vs*.* 1/7	2/7 vs*.* 1/7
N (eyes)	47	49	42				
N per sex (eyes)							
Male	20	22	20	0.891			
Female	27	27	22				
Age (years)	10.62 ± 1.88	10.18 ± 1.94	10.60 ± 2.13	0.488			
Baseline SE (D)	− 2.38 ± 1.22	− 2.19 ± 1.00	− 2.19 ± 1.12	0.646			
∆SE (D)	− 0.13 ± 0.55	− 0.21 ± 0.47	− 0.55 ± 0.46	< 0.001	1.000	< 0.001	0.004
Baseline AL (mm)	24.57 ± 0.80	24.36 ± 0.86	24.37 ± 0.71	0.348			
∆AL (mm)	0.09 ± 0.23	0.17 ± 0.17	0.26 ± 0.20	0.001	0.172	< 0.001	0.098

*N* = number of eyes; *SE* = spherical equivalent; ∆*SE* = one year SE minus baseline SE; *AL* = axial length; ∆*AL* = one year AL minus baseline AL. 7/7 Group = once-daily group; 2/7 Group = twice per week group; 1/7 Group = once per week group. ^#^One way ANOVA

Figure 2 illustrates comparisons among the three groups, notably emphasizing the 7/7 group, where a segment of the population showed AL regression and reduction in SE. Such occurrences decreased sequentially in the 2/7 and 1/7 groups.

### PR and RPR changes among groups

**Fig. 3:**
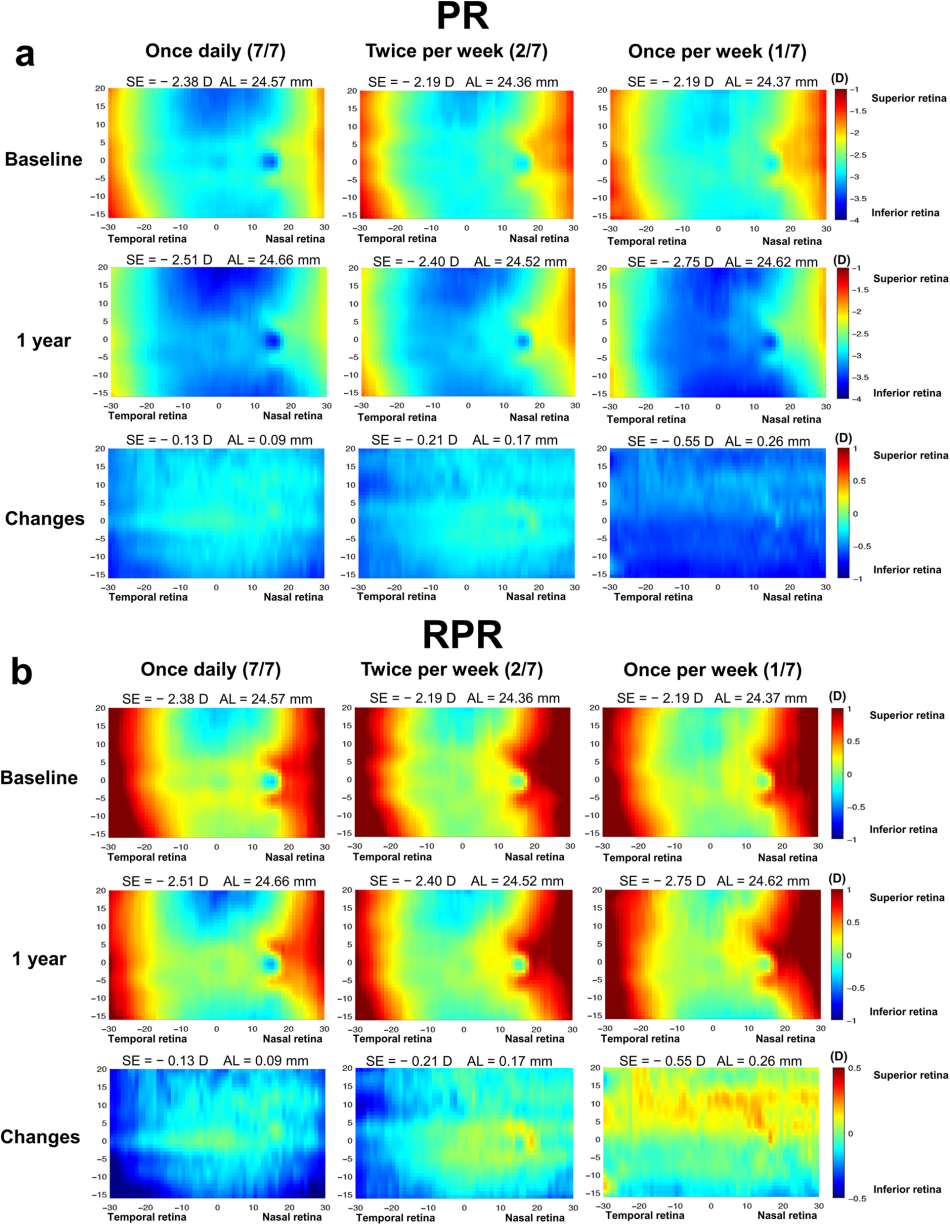
Two-dimensional (2D) maps of peripheral refraction (PR) and relative peripheral refraction (RPR) with 0.05% atropine eyedrop usage at different dosage regimens in a one-year period. **a** PR at baseline, one year, and changes (one year minus baseline); **b** RPR at baseline, one year, and changes (one year minus baseline). On the x-axis, positive and negative values indicate the nasal-retinal and temporal-retinal areas, respectively, and those on the y-axis represent the superior and inferior retinal-areas, respectively. SE, spherical equivalent; AL, axial length

Figure 3 depicts the 2D refraction maps for the different groups and measurement times, spanning from baseline to the one-year follow-up. PR, RPR, and one-year changes among groups are shown in Fig. 3, and detailed in Additional files 3 and 4. Statistically significant differences in PR changes emerged among the three groups in vertical and nasal directions of the retina (regions S, Fovea, N, I, and IN). Post hoc analyses revealed distinctions between the 1/7 group and the other two groups (all *P* < 0.05). Regarding RPR at the one-year time point, significant differences between the 7/7 and 1/7 groups were observed in the S and fovea regions (all *P* < 0.05). However, no significant differences were found in other regions among the groups. Furthermore, RPR changes among the three groups did exhibit significant differences in the superior, nasal, and temporal regions of the periphery retina (regions ST, S, SN, T, N, IT, and IN). Post hoc analyses indicated differences between the 1/7 group and the other two groups (all *P* < 0.05).

## Longitudinal changes in RPR across retinal regions in each group

**Fig. 4:**
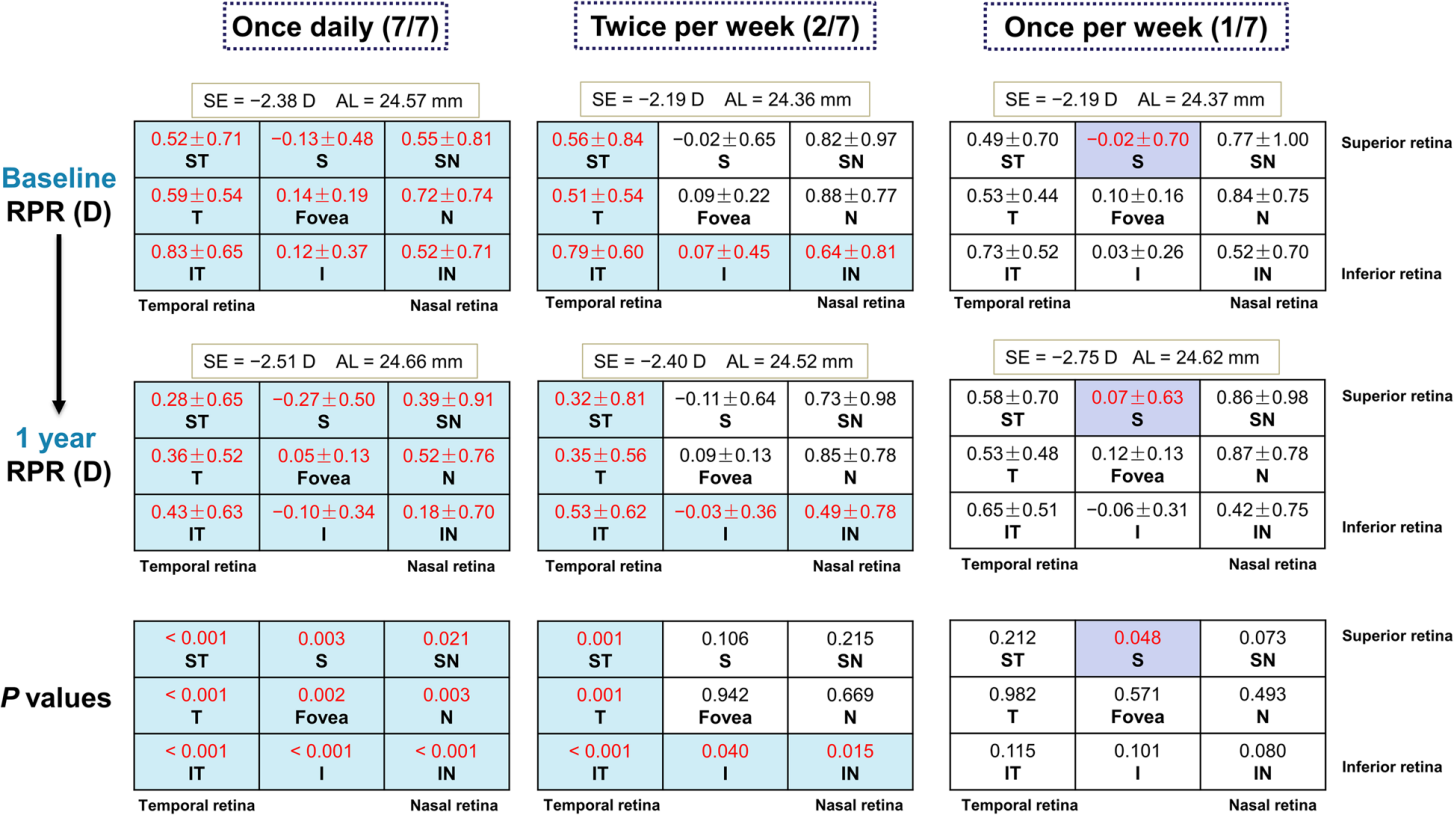
Longitudinal comparison of relative peripheral refraction (RPR) at baseline and one year, with corresponding *P* values for each group. Mean ± SD of RPR in nine regions (ST: superior temporal, S: superior, SN: superior nasal, T: temporal, Fovea, N: nasal, IT: inferior temporal, I: inferior, and IN: inferior nasal). SE, spherical equivalent; AL, axial length. Values in red indicate that the changes in RPR before and after one year are statistically significant

At baseline, only RPR for the S zone showed myopic defocus in each group, while RPR for all other zones indicated hyperopic defocus, as detailed in Fig. 4. After one year of 0.05% atropine administration, the 7/7 group displayed a significant myopic shift in RPR across all regions of the retina (all *P* < 0.05). In the 2/7 group, RPR showed a significant myopic shift in the temporal and inferior retina (regions ST, T, IT, I, and IN; all *P* < 0.05). Conversely, in the 1/7 group, RPR showed a significant hyperopic shift in the superior retina (region S, *P* = 0.048), with no significant differences found in other regions.

### Correlation of RPR changes with myopia progression and AL changes in each group

**Fig. 5:**
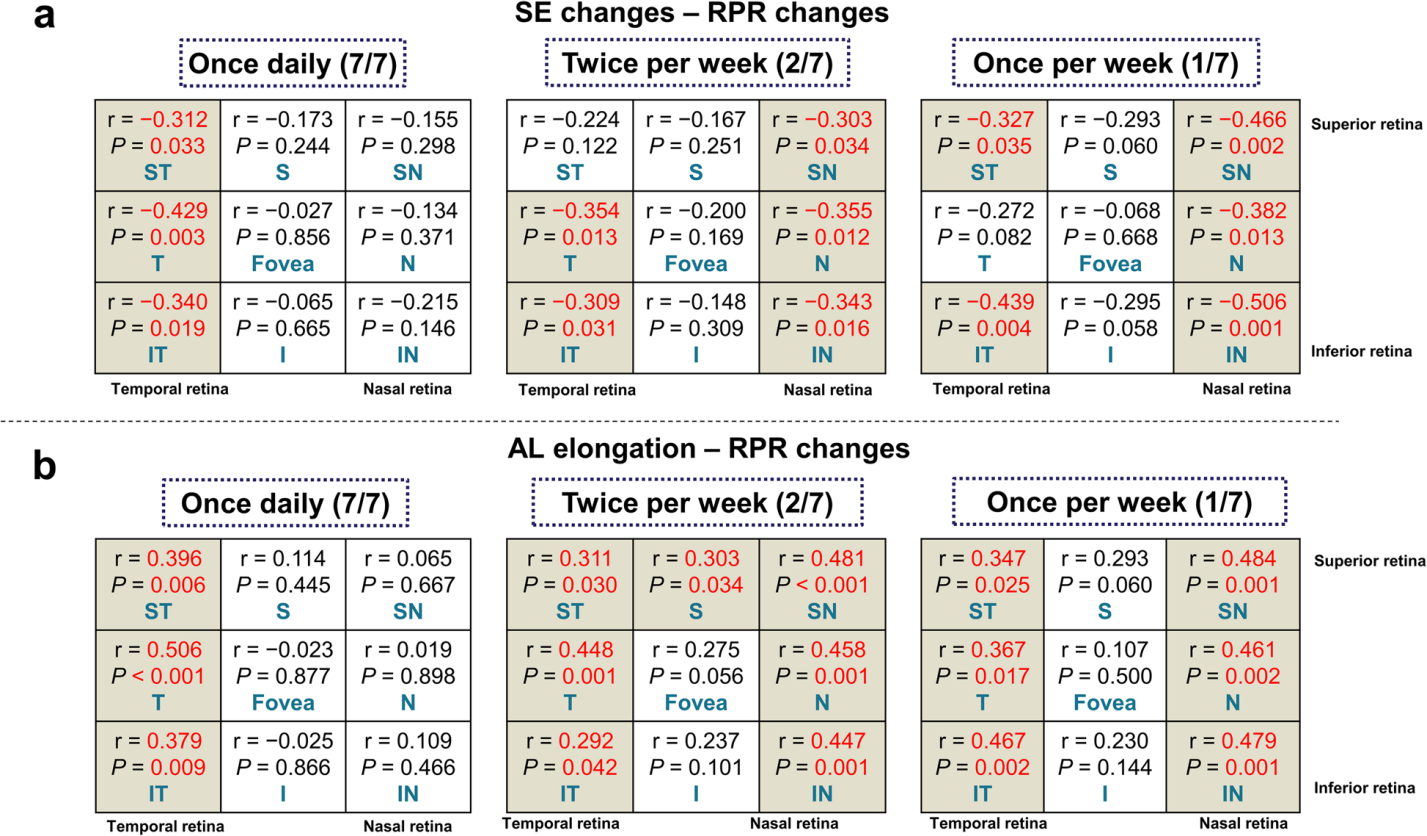
Correlation analysis of relative peripheral refraction (RPR) changes with myopia progression and axial length (AL) changes in each group. SE, spherical equivalent; ST, superior temporal; S, superior; SN, superior nasal; T, temporal; N, nasal; IT, inferior temporal; I, inferior; IN, inferior nasal. Values in red indicate that the changes in SE or AL are statistically significantly correlated with changes in RPR

The correlation of changes in RPR with myopia progression, as well as AL changes, was analyzed for each group. Specific RPR regions that correlated significantly with SE changes also demonstrated significant correlations with AL changes, for the most part (Fig. 5). In the 7/7 group, RPR changes at the temporal peripheral retina (ST, T, and IT) showed a notable correlation with myopia progression and AL changes (all *P* < 0.05). Within the 2/7 group, RPR changes at the nasal and temporal peripheral retina (SN, T, N, IT, and IN) showed a significant association with myopia progression, while RPR changes at the superior, nasal and temporal peripheral retina (ST, S, SN, T, N, IT, and IN) showed a significant association with AL changes (all *P* < 0.05). Similarly, in the 1/7 group, RPR changes at the nasal and temporal peripheral retina (ST, SN, N, IT, and IN) showed a significant association with myopia progression, and RPR changes at the nasal and temporal peripheral retina (ST, SN, T, N, IT, and IN) showed a significant association with AL changes (all *P* < 0.05).

**Fig. 6:**
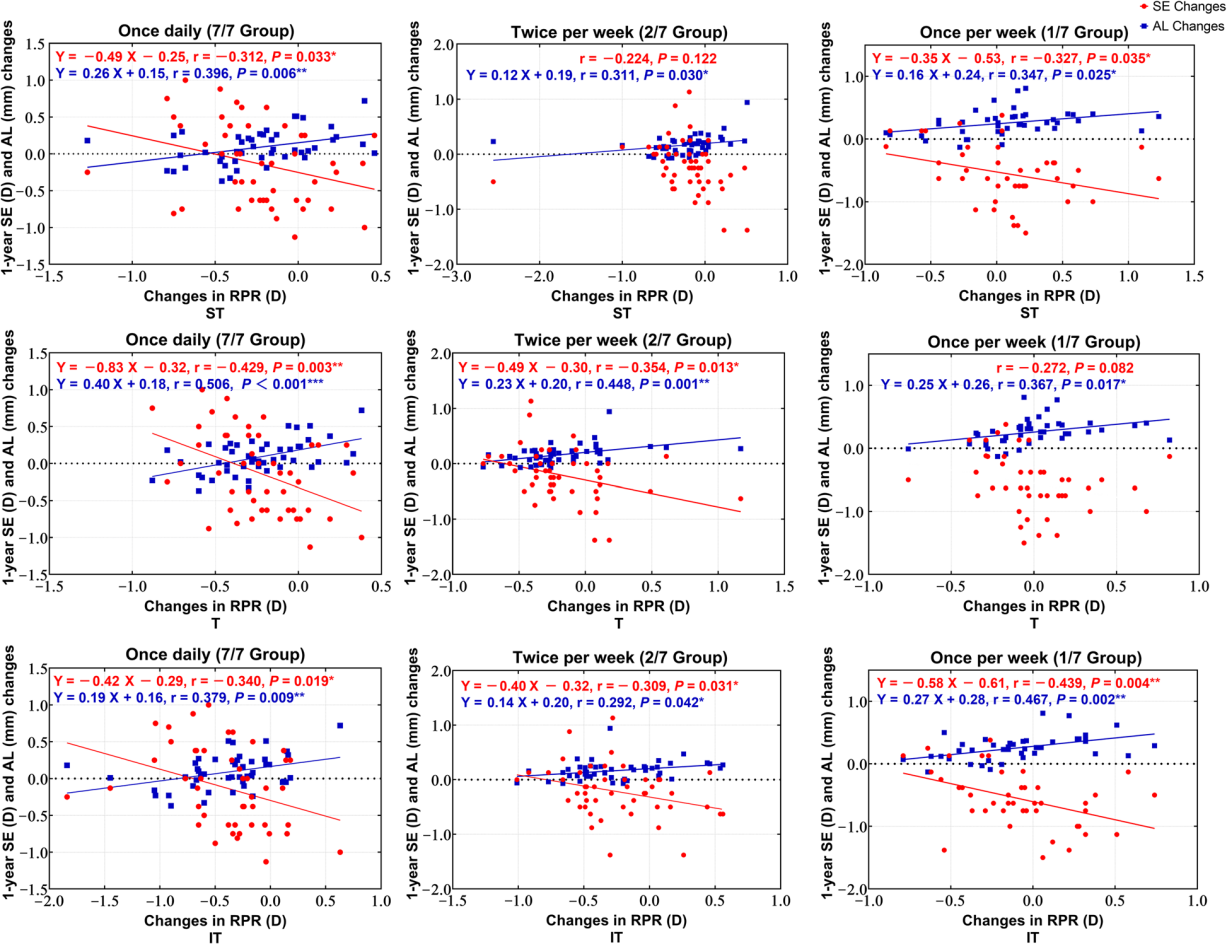
Correlation of changes in relative peripheral refraction (RPR) with SE and AL changes in the temporal retina for each group. SE, spherical equivalent; AL, axial length; ST, superior temporal; T, temporal; IT, inferior temporal. *** *P* < 0.001, ** *P* < 0.01, * *P* < 0.05

Figure 6 describes the correlation between RPR changes with SE and AL changes in the temporal retina for each group. More myopic shift of RPR in the temporal peripheral retina was associated with less myopia progression and fewer AL changes (all *P* < 0.05). All three groups demonstrated varying degrees of this same regional correlation in the temporal peripheral retina, notably the 7/7 group. In the plots corresponding to the 7/7 group, some individuals exhibit AL regression and SE reduction, where the change in the AL is negative and the change in SE is positive. Additionally, the correlation lines intersect at the y-axis at the 0 scale, indicating that the SE and AL changes have a point of intersection at y = 0. Furthermore, it becomes apparent that the AL regression and SE reduction also increase with a greater myopic shift in RPR.

## Discussion

This longitudinal study investigated the effects of a year-long administration of 0.05% atropine eyedrop at three different frequencies for myopia control, specifically examining the evolution of 2D PR and RPR in myopic children. The 7/7 group demonstrated the largest treatment effect. In line with the LAMP study of 0.05% concentration, the 7/7 group in this study showed yearly myopia progression (− 0.27 ± 0.61 D vs. − 0.13 ± 0.55 D) and axial elongation (0.20 ± 0.25 mm vs. 0.09 ± 0.23 mm) for myopia control [6]. These findings collectively suggest efficacy in managing myopia progression. Additionally, the 1/7 group exhibited yearly myopia progression (− 0.55 ± 0.46 D) similar to the LAMP (− 0.59 ± 0.61 D) and ATOM2 study (− 0.43 ± 0.52 D) involving a 0.01% concentration for myopia control [6, 23]. Similar to Li et al., our study found that some individuals in the 7/7 group experienced AL and SE reductions, with decreases correlating with reduced atropine usage frequency [24]. Additionally, we hypothesized that AL retraction may be related to choroidal changes as indicated by the concentration-dependent choroidal thickening effect associated with alterations in SE and AL [25]. 0.05% atropine eyedrop demonstrated frequency-dependent inhibition of myopia progression in this study, with the strongest effect observed with once-daily usage.

Notably, this study revealed varying degrees of myopia progression, as well as variations in PR and RPR among the three frequency groups. The 1/7 group demonstrated greater myopic PR in the vertical and nasal retina compared to the other groups. Atchison et al. revealed myopia progression, as observed through magnetic resonance imaging (MRI), resulting in a sequential increase in ellipsoid dimensions: axial > vertical > horizontal [26]. The 7/7 group not only exhibited reduced axial elongation but also demonstrated more myopic RPR in the vertical dimension after one year, particularly in the fovea and superior retina. The two higher frequency groups experienced more myopic shifts in RPR in the peripheral retina. As myopia progresses, myopic children's eyes elongate and become less oblate, with the axial dimension growing the most, followed by the vertical and horizontal dimensions, in a ratio of approximately 3:2:1 [27]. RPR changes may indirectly describe retinal shape and reflect different patterns of eye growth [28]. The 1/7 group elongated the most and displayed less oblateness in retinal morphology, while the 7/7 and 2/7 groups tended towards oblateness in the retina. The potent myopia inhibition induced by atropine has effectively reversed the trend of ocular elongation and decreased oblateness observed in myopic children's eyes [29–31]. Different frequencies of atropine use exert varying effects on myopic control and induce distinct changes in peripheral defocus.

In terms of longitudinal comparison within each group, the 7/7 group exhibited more myopic shifts across the entire retinal extent than the 2/7 group in the temporal and inferior retina, while the 1/7 group displayed a hyperopic shift in the superior retina. Children in the once-daily atropine group exhibited the least myopia progression and a more myopic shift in RPR. Similar changes in defocus were observed during orthokeratology wear with significant RPR changes towards a more myopic defocus occurring from baseline to the six-month follow-up across all visual fields [32]. Wearing defocus incorporated multiple segments (DIMS) lenses induced a uniform RPR myopic shift along the horizontal retina, demonstrating notable myopia control effects compared to single-vision lenses [29]. Atropine use resulted in various RPR changes across different areas and degrees of peripheral retina. Animal experiments suggest that the impact of vision on refractive development in primates is dominated by local retinal mechanisms, integrating visual signals in a spatially restricted manner and exerting their influence selectively on the subjacent sclera [33, 34]. Atropine may enhance the neuronal responses to myopic defocus in the inner layers of the peripheral retina, and thus potentiate the effects of myopic defocus in inhibiting eye growth [35]. Choroidal thickness shows bi-directional changes in response to the sign of defocus, and the eye detects the type of retinal blur [25, 36]. Regional variations in RPR changes may be associated with local retinal mechanisms and changes in choroidal thickness. Further exploration of the correlation between peripheral defocus and choroidal changes could yield valuable insights.

A trend was noted across all groups, suggesting a potential association between the temporal retina and myopia progression. Although a correlation between the nasal retina and changes in SE and AL was observed in the lower-frequency group, it is noteworthy that the RPR changes in the nasal retina were not significant across all groups. In the 7/7 group, the most effective for myopia control, a more myopic RPR or a less hyperopic RPR in the temporal retina significantly correlated with reduced myopia progression despite the concurrent presence of AL regression and SE reduction. These findings imply that alterations in the growth of one retinal region may exert influence over the growth of another. Lin et al. indicated relative myopic defocus in the superior retina as a predictor of central myopia shift in emmetropic children [37]. Myopic children undergoing orthokeratology with temporal lens decentration exhibited better myopia control, with myopic defocus significantly increasing in the temporal retina [22]. Animal experiments indicated that, in terms of decoding optical input for growth, the area of the retina exposed to optical signals may be critical in determining eye growth [38]. In general, it is unlikely for ocular growth to bring the image simultaneously into focus across the full field. Ocular elongation may rely on a weighted average of defocus signals from various parts of the retina or retinal visual signaling [39]. The BLINK study suggests that wearing multifocal contact lenses with a + 2.50 D addition can reverse the increase in retinal steepness induced by single-vision lenses, indicating that optical myopia therapy may involve spatial integration or mechanisms beyond local defocus [31]. This study suggests a potential correlation between defocus in the temporal retinal region and myopia progression, further confirmation of this hypothesis is required.

Our investigation on peripheral defocus and atropine not only reaffirms the changes in defocus observed in previous studies following atropine administration but also elucidates variations in defocus changes under different dosing regimens. The implications of this study are crucial for ophthalmologists and patient education, highlighting the importance of daily dosage and the necessity for compliance. We propose integrating changes in peripheral defocus assessments with atropine in clinical practice to evaluate or predict myopia control outcomes. The study's limitation lies in the absence of a placebo control group and the lack of cycloplegia in PR. Additionally, the once-daily group, commonly used clinically, was designated as the reference group. Further investigation into the relationship between peripheral defocus and myopia progression after longer-term atropine eyedrops usage in a larger sample of children is therefore warranted.

## Conclusions

0.05% atropine eyedrops effectively inhibit myopia progression in a frequency-dependent manner. Importantly, the once-daily group showed the slowest myopia progression but exhibited more myopic shifts in RPR. Additionally, RPR in the temporal retina was related to myopia progression in all groups.

### Supplementary Information

Additional file 1. Flow diagram of participants in this study.

Additional file 2. Detailed statistical methods employed in this study.

Additional file 3. Peripheral refraction (PR) among the three different atropine dosage groups at the one-year interval.

Additional file 4. Relative Peripheral refraction (RPR) among the three different atropine dosage groups at the one-year interval.

## Data Availability

The datasets used and/or analyzed during the current study are available from the corresponding author upon reasonable request.

## Abbreviations

SE: Spherical equivalent
AL: Axial length
PR: Peripheral refraction
RPR: Relative peripheral refraction
2D: Two-dimensional
