# Supplementary figures and images for: Frequency-dependent effects of 0.05% atropine eyedrops on myopia progression and peripheral defocus: a prospective study
Source: Eye Vis (Lond). 2024 Aug 1;11:26. doi: 10.1186/s40662-024-00395-0 (PMC11293060; doi:10.1186/s40662-024-00395-0)

**Additional File 1**

**Figure S1.** Flow diagram of participants in the present study.


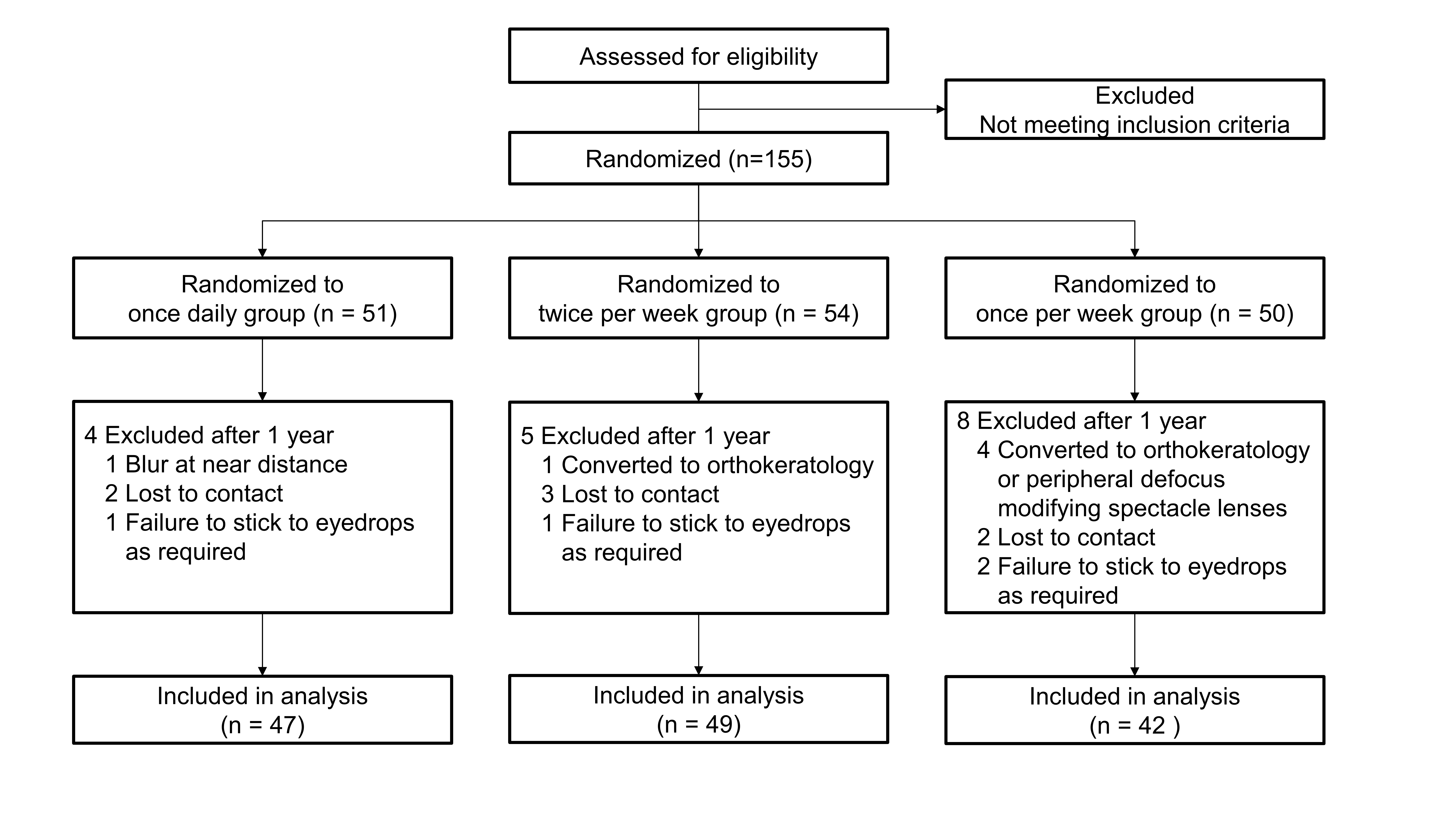

Supplement: Supplementary file 1 — Additional file 1. Flow diagram of participants in this study. [file 40662_2024_395_MOESM1_ESM.docx]
