# Supplementary material for: Frequency-dependent effects of 0.05% atropine eyedrops on myopia progression and peripheral defocus: a prospective study
Source: Eye Vis (Lond). 2024 Aug 1;11:26. doi: 10.1186/s40662-024-00395-0 (PMC11293060; doi:10.1186/s40662-024-00395-0)
Supplement: Supplementary file 2 — Additional file 2. Detailed statistical methods employed in this study. [file 40662_2024_395_MOESM2_ESM.docx]

**Additional File 2**

**Table S1.** Detailed statistical methods employed in the study

| **Hypothesis** | **Test** |
| --- | --- |
| Comparison of age, SE, AL, PR, RPR, and one-year change among groups | One-way ANOVA |
| Post hoc analysis for multiple comparisons | Bonferroni test |
| Sex distribution differences | Chi-square test |
| One-year longitudinal changes in RPR within each group of different retinal areas | Paired t-test |
| Relationships between RPR changes in each zone and changes in SE or AL | Pearson’s correlation |

SE = spherical equivalent; AL = axial length; PR = peripheral refraction; RPR = relative peripheral refraction; ANOVA = analysis of variance
