# Supplementary material for: Frequency-dependent effects of 0.05% atropine eyedrops on myopia progression and peripheral defocus: a prospective study
Source: Eye Vis (Lond). 2024 Aug 1;11:26. doi: 10.1186/s40662-024-00395-0 (PMC11293060; doi:10.1186/s40662-024-00395-0)
Supplement: Supplementary file 3 — Additional file 3. Peripheral refraction (PR) among the three different atropine dosage groups at the one-year interval. [file 40662_2024_395_MOESM3_ESM.docx]

**Additional File** **3**

**Table S2**. Peripheral refraction (PR) among the three different atropine dosage groups at one-year interval

| **Baseline** | | | | | | | | |
| --- | --- | --- | --- | --- | --- | --- | --- | --- |
| Region | 7/7 Group | 2/7 Group | 1/7 Group | *P** | Post hoc | | | |
| SE | −2.38 ± 1.22 | −2.19 ± 1.00 | −2.19 ± 1.12 | 0.646 | - | - | - | |
| AL | 24.57 ± 0.80 | 24.36 ± 0.86 | 24.37 ± 0.71 | 0.348 | - | - | - | |
| ST | −2.50 ± 1.33 | −2.31 ± 0.87 | −2.35 ± 1.20 | 0.701 | - | - | - | |
| S | −3.15 ± 1.22 | −2.90 ± 0.84 | −2.87 ± 1.28 | 0.409 | - | - | - | |
| SN | −2.48 ± 1.25 | −2.06 ± 1.00 | −2.08 ± 1.34 | 0.172 | - | - | - | |
| T | −2.43 ± 1.25 | −2.37 ± 0.90 | −2.32 ± 1.04 | 0.881 | - | - | - | |
| Fovea | −2.89 ± 1.17 | −2.79 ± 0.94 | −2.75 ± 1.08 | 0.808 | - | - | - | |
| N | −2.30 ± 1.23 | −2.00 ± 1.00 | −2.01 ± 1.20 | 0.352 | - | - | - | |
| IT | −2.20 ± 1.20 | −2.09 ± 0.89 | −2.12 ± 1.04 | 0.874 | - | - | - | |
| I | −2.91 ± 1.18 | −2.81 ± 1.01 | −2.82 ± 1.09 | 0.887 | - | - | - | |
| IN | −2.50 ± 1.25 | −2.23 ± 1.05 | −2.32 ± 1.18 | 0.514 | - | - | - | |
| **One year** | | | | | | | | |
| Region | 7/7 Group | 2/7 Group | 1/7 Group | *P** |  |  | |  |
| SE | −2.51±1.38 | −2.40±1.06 | −2.75±1.24 | 0.398 | - | - | - | |
| AL | 24.66±0.79 | 24.52±0.85 | 24.62±0.70 | 0.692 | - | - | - | |
| ST | −2.87±1.44 | −2.76±0.98 | −2.84±1.33 | 0.911 | - | - | - | |
| S | −3.42±1.34 | −3.20±0.98 | −3.35±1.36 | 0.662 | - | - | - | |
| SN | −2.76±1.55 | −2.36±1.14 | −2.56±1.54 | 0.382 | - | - | - | |
| T | −2.79±1.35 | −2.74±0.97 | −2.89±1.17 | 0.837 | - | - | - | |
| Fovea | −3.10±1.27 | −2.99±1.02 | −3.30±1.17 | 0.448 | - | - | - | |
| N | −2.63±1.48 | −2.24±1.10 | −2.55±1.40 | 0.325 | - | - | - | |
| IT | −2.72±1.35 | −2.55±0.98 | −2.77±1.06 | 0.630 | - | - | - | |
| I | −3.25±1.29 | −3.12±1.07 | −3.48±1.04 | 0.321 | - | - | - | |
| IN | −2.97±1.44 | −2.60±1.12 | −3.00±1.28 | 0.230 | - | - | - | |
| **Changes** | | | | | | | | |
| Region | 7/7 Group | 2/7 Group | 1/7 Group | *P** | 7/7 vs. 2/7 Group | 7/7 vs. 1/7 Group | 2/7 vs. 1/7 Group | |
| SE | −0.13 ± 0.55 | −0.21 ± 0.47 | −0.55 ± 0.46 | < 0.001 | 1.000 | < 0.001 | 0.004 | |
| AL | 0.09 ± 0.23 | 0.17 ± 0.17 | 0.26 ± 0.20 | 0.001 | 0.172 | < 0.001 | 0.098 | |
| ST | −0.37 ± 0.40 | −0.45 ± 0.31 | −0.49 ± 0.48 | 0.364 | - | - | - | |
| S | −0.27 ± 0.42 | −0.30 ± 0.30 | −0.48 ± 0.41 | 0.021 | 1.000 | 0.027 | 0.08 | |
| SN | −0.28 ± 0.54 | −0.30 ± 0.34 | −0.48 ± 0.36 | 0.061 | - | - | - | |
| T | −0.36 ± 0.38 | −0.38 ± 0.41 | −0.57 ± 0.46 | 0.033 | 1.000 | 0.054 | 0.081 | |
| Fovea | −0.21 ± 0.44 | −0.21 ± 0.49 | −0.56 ± 0.45 | < 0.001 | 1.000 | 0.002 | 0.001 | |
| N | −0.33 ± 0.56 | −0.24 ± 0.57 | −0.54 ± 0.40 | 0.025 | 1.000 | 0.180 | 0.023 | |
| IT | −0.52 ± 0.38 | −0.46 ± 0.44 | −0.66 ± 0.43 | 0.082 | - | - | - | |
| I | −0.34 ± 0.46 | −0.31 ± 0.48 | −0.66 ± 0.45 | 0.001 | 1.000 | 0.005 | 0.002 | |
| IN | −0.47 ± 0.49 | −0.36 ± 0.50 | −0.68 ± 0.40 | 0.007 | 0.779 | 0.120 | 0.005 | |

SE = spherical equivalent; AL = axial length; ST = superior temporal; S = superior; SN = superior nasal; T = temporal; N = nasal; IT = inferior temporal; I = inferior; IN = inferior nasal.

PR values present as mean ± SD at baseline, one year, and changes (one year minus baseline) in the corresponding region.

*Indicates the statistics among the three different frequency groups (7/7 Group: once daily, 2/7 Group: twice per week, 1/7 Group: once per week). Statistical significance was set at 0.05. A 95% confidence interval (CI) is shown for significance for the difference in means at *P* < 0.05 (with Post hoc analysis for multiple comparisons conducted using Bonferroni tests).
