# Supplementary material for: Frequency-dependent effects of 0.05% atropine eyedrops on myopia progression and peripheral defocus: a prospective study
Source: Eye Vis (Lond). 2024 Aug 1;11:26. doi: 10.1186/s40662-024-00395-0 (PMC11293060; doi:10.1186/s40662-024-00395-0)
Supplement: Supplementary file 4 — Additional file 4. Relative Peripheral refraction (RPR) among the three different atropine dosage groups at the one-year interval. [file 40662_2024_395_MOESM4_ESM.docx]

**Additional File 4**

**Table S3.** Relative peripheral refraction (RPR) among the three different atropine dosage groups at one-year interval

| **Baseline** | | | | | | | |
| --- | --- | --- | --- | --- | --- | --- | --- |
| Region | 7/7 Group | 2/7 Group | 1/7 Group | *P** | Post hoc | | |
| SE | −2.38 ± 1.22 | −2.19 ± 1.00 | −2.19 ± 1.12 | 0.646 | - | - | - |
| AL | 24.57 ± 0.80 | 24.36 ± 0.86 | 24.37 ± 0.71 | 0.348 | - | - | - |
| ST | 0.52 ± 0.71 | 0.56 ± 0.84 | 0.49 ± 0.70 | 0.908 | - | - | - |
| S | −0.13 ± 0.48 | −0.02 ± 0.65 | −0.02 ± 0.70 | 0.613 | - | - | - |
| SN | 0.55 ± 0.81 | 0.82 ± 0.97 | 0.77 ± 1.00 | 0.328 | - | - | - |
| T | 0.59 ± 0.54 | 0.51 ± 0.54 | 0.53 ± 0.44 | 0.716 | - | - | - |
| Fovea | 0.14 ± 0.19 | 0.09 ± 0.22 | 0.10 ± 0.16 | 0.495 | - | - | - |
| N | 0.72 ± 0.74 | 0.88 ± 0.77 | 0.84 ± 0.75 | 0.578 | - | - | - |
| IT | 0.83 ± 0.65 | 0.79 ± 0.60 | 0.73 ± 0.52 | 0.755 | - | - | - |
| I | 0.12 ± 0.37 | 0.07 ± 0.45 | 0.03 ± 0.26 | 0.531 | - | - | - |
| IN | 0.52 ± 0.71 | 0.64 ± 0.81 | 0.52 ± 0.70 | 0.653 | - | - | - |
| **One year** | | | | | | | |
| Region | 7/7 Group | 2/7 Group | 1/7 Group | *P** | 7/7 vs. 2/7 Group | 7/7 vs. 1/7 Group | 2/7 vs. 1/7 Group |
| SE | −2.51 ± 1.38 | −2.40 ± 1.06 | −2.75 ± 1.24 | 0.398 | - | - | - |
| AL | 24.66 ± 0.79 | 24.52 ± 0.85 | 24.62 ± 0.70 | 0.692 | - | - | - |
| ST | 0.28 ± 0.65 | 0.32 ± 0.81 | 0.58 ± 0.70 | 0.114 | - | - | - |
| S | −0.27 ± 0.50 | −0.11 ± 0.64 | 0.07 ± 0.63 | 0.029 | 0.556 | 0.024 | 0.468 |
| SN | 0.39 ± 0.91 | 0.73 ± 0.98 | 0.86 ± 0.98 | 0.057 | - | - | - |
| T | 0.36 ± 0.52 | 0.35 ± 0.56 | 0.53 ± 0.48 | 0.183 | - | - | - |
| Fovea | 0.05 ± 0.13 | 0.09 ± 0.13 | 0.12 ± 0.13 | 0.045 | 0.297 | 0.045 | 1.000 |
| N | 0.52 ± 0.76 | 0.85 ± 0.78 | 0.87 ± 0.78 | 0.053 | - | - | - |
| IT | 0.43 ± 0.63 | 0.53 ± 0.62 | 0.65 ± 0.51 | 0.248 | - | - | - |
| I | −0.10 ± 0.34 | −0.03 ± 0.36 | −0.06 ± 0.31 | 0.589 | - | - | - |
| IN | 0.18 ± 0.70 | 0.49 ± 0.78 | 0.42 ± 0.75 | 0.098 | - | - | - |
| **Changes** | | | | | | | |
| Region | 7/7 Group | 2/7 Group | 1/7 Group | *P** | 7/7 vs. 2/7 Group | 7/7 vs. 1/7 Group | 2/7 vs. 1/7 Group |
| SE | −0.13 ± 0.55 | −0.21 ± 0.47 | −0.55 ± 0.46 | < 0.001 | 1.00 | < 0.001 | 0.004 |
| AL | 0.09 ± 0.23 | 0.17 ± 0.17 | 0.26 ± 0.20 | 0.001 | 0.172 | < 0.001 | 0.098 |
| ST | −0.24 ± 0.35 | −0.24 ± 0.45 | 0.09 ± 0.44 | < 0.001 | 1.00 | 0.001 | 0.001 |
| S | −0.14 ± 0.31 | −0.09 ± 0.39 | 0.09 ± 0.29 | 0.003 | 1.00 | 0.004 | 0.030 |
| SN | −0.16 ± 0.45 | −0.08 ± 0.47 | 0.10 ± 0.34 | 0.019 | 1.00 | 0.018 | 0.138 |
| T | −0.23 ± 0.28 | −0.16 ± 0.34 | 0.00 ± 0.30 | 0.002 | 0.827 | 0.001 | 0.035 |
| Fovea | −0.09 ± 0.18 | 0.00 ± 0.27 | 0.02 ± 0.19 | 0.051 | - | - | - |
| N | −0.20 ± 0.43 | −0.03 ± 0.48 | 0.04 ± 0.33 | 0.026 | 0.152 | 0.029 | 1.000 |
| IT | −0.39 ± 0.44 | −0.25 ± 0.36 | −0.09 ± 0.35 | 0.001 | 0.241 | 0.001 | 0.13 |
| I | −0.22 ± 0.38 | −0.10 ± 0.33 | −0.08 ± 0.33 | 0.148 | - | - | - |
| IN | −0.34 ± 0.47 | −0.15 ± 0.42 | −0.11 ± 0.38 | 0.019 | 0.083 | 0.028 | 1.000 |

SE = spherical equivalent; AL = axial length; ST = superior temporal; S = superior; SN = superior nasal; T = temporal; N = nasal; IT = inferior temporal; I = inferior; IN = inferior nasal.

RPR values present as mean ± SD at baseline, one year, and changes (one year minus baseline) in the corresponding region.

*Indicates the statistics among the three different frequency groups (7/7 Group: once daily, 2/7 Group: twice per week, 1/7 Group: once per week). Statistical significance was set at 0.05. A 95% confidence interval (CI) is shown for significance for the difference in means at *P* < 0.05 (with Post hoc analysis for multiple comparisons conducted using Bonferroni tests).
